# Supplementary material for: Spin mapping of intralayer antiferromagnetism and field-induced spin reorientation in monolayer CrTe2
Source: Nat Commun. 2022 Jan 11;13:257. doi: 10.1038/s41467-021-27834-z (PMC8752801; doi:10.1038/s41467-021-27834-z)
Supplement: Supplementary file 1 — Supplementary Information [file 41467_2021_27834_MOESM1_ESM.docx]

**Supplementary information for**

**Spin mapping of intralayer antiferromagnetism and field-induced spin reorientation in monolayer CrTe_2_**

Jing-Jing Xian^1†^, Cong Wang^2†^, Jin-Hua Nie^1†^, Rui Li^1^, Mengjiao Han^3, 4^, Junhao Lin^3, 4^, Wen-Hao Zhang^1^, Zhen-Yu Liu^1^, Zhi-Mo Zhang^1^, Mao-Peng Miao^1^, Yangfan Yi^5^, Shiwei Wu^5^, Xiaodie Chen^1^, Junbo Han^1^, Zhengcai Xia^1^, Wei Ji^2#^, Ying-Shuang Fu^1*^

1. School of Physics and Wuhan National High Magnetic Field Center, Huazhong University of Science and Technology, Wuhan 430074, China
2. Beijing Key Laboratory of Optoelectronic Functional Materials and Micro-Nano Devices, Department of Physics, Renmin University of China, Beijing 100872, China
3. Department of Physics, Southern University of Science and Technology, Shenzhen 518055, China
4. Shenzhen Key Laboratory for Advanced Quantum Functional Materials and Devices, Southern University of Science and Technology, Shenzhen 518055, China
5. State Key Laboratory of Surface Physics, Department of Physics, Fudan University, Shanghai 200433, China

^†^These authors contributed equally to this work.

Emails: ^#^[wji@ruc.edu.cn](mailto:wji@ruc.edu.cn), [*yfu@hust.edu.cn](mailto:*yfu@hust.edu.cn)

**SUPPLEMENTARY NOTES**

**Magnetization orientations of Cr tip and CrTe_2_.**

The magnetization orientations of the Cr tip and CrTe_2_ are determined in the following way. First, from Fig. S9, the spin contrast varies at different domains of CrTe_2_ at zero magnetic field. This suggests both the tip and sample have in-plane component of magnetization. Second, the zigzag spin contrast reverses its phase under a small external field of 0.2 T (Fig. S6), that is applied perpendicular to the basal plane of CrTe_2_. This demonstrates both the tip and sample should have out-of-plane component of magnetization at 0.2 T. The Cr tip magnetization hardly changes under small magnetic fields, whose switching threshold field is typically an order of magnitude larger, due to the antiferromagnetism of the Cr tip with strong magnetic anisotropy [S1]. Therefore, the observed spin contrast reversal comes from the sample, rather than the tip. We can also clearly evaluate whether the tip magnetization was preserved during the measurement. If the tip magnetization switches, the observed zigzag spin pattern would reverse its contrast abruptly with increasing magnetic field, which was not observed in Fig. S6. Moreover, the zigzag spin pattern recovers to its original contrast once the magnetic field is removed. All this information rigorously demonstrates the tip magnetization was preserved. Third, the magnetization of the CrTe_2_ is not purely in-plane at zero magnetic field. Otherwise, one would expect the same phase of spin contrast between the ±0.2 T. Thus, the magnetization orientation proposed in Fig. 3h is the most plausible scenario.

The magnetization orientation of CrTe_2_ is also substantiated from the measurement with Fe-coated tips. The Fe-coated tip has in-plane magnetization at zero magnetic field, because of the surface and interface anisotropy of the coated ferromagnetic Fe film [S1-S3]. The spin contrast of different domains also varies in Fig. S10a, confirming the in-plane spin component of CrTe_2_. The Fe tip magnetization can be aligned to out-of-plane with a magnetic field applied perpendicular to the basal plane. Under a 3 T out-of-plane filed, the spin contrast at the upper-right domain of Fig. Fig. S10b has similar zig-zag spin contrast as that of the lower-left domain. This conforms to the out-of-plane spin component of CrTe_2_. The spin contrast of Fig. S10a recovers once the magnetic field is removed, demonstrating the magnetization of the Fe tip recovers to its original direction.

**SUPPLEMENTARY FIGURES**


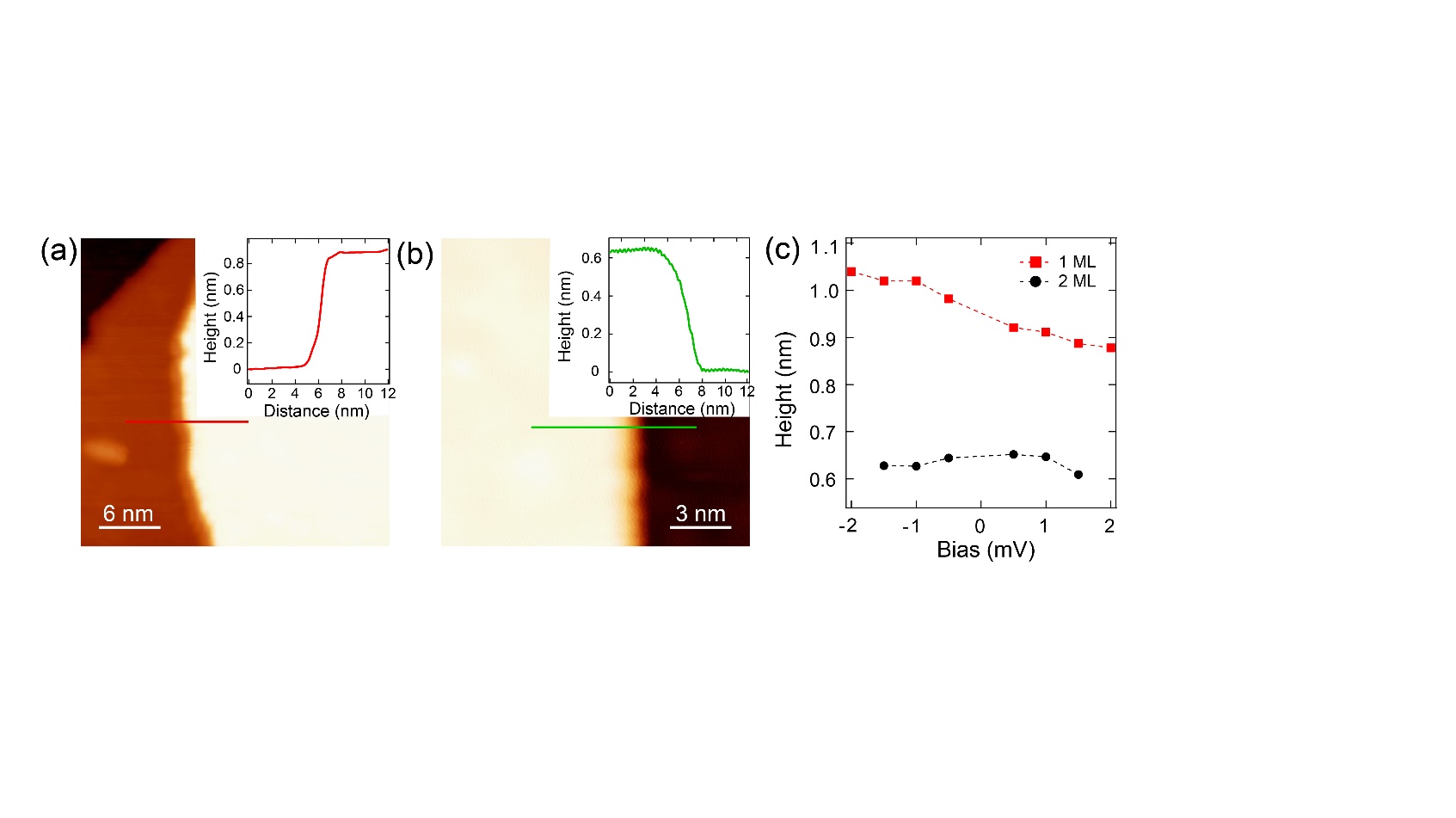


**Figure S1 ┃Bias-dependent apparent height of 1 ML and 2 ML 1T-CrTe_2_ films.** (a,b) STM images of 1 ML (a) and 2 ML (b) 1T-CrTe_2_ films on the graphene substrate. The insets show line profiles along the red (*V*_b_ = +2 V, *I*_t_ = 10 pA) and green lines (*V*_b_ = +1.5 V, *I*_t_ = 10 pA). (c) Apparent height of 1 ML (red) and 2 ML (black) 1T-CrTe_2_ films at different tunnelling biases.


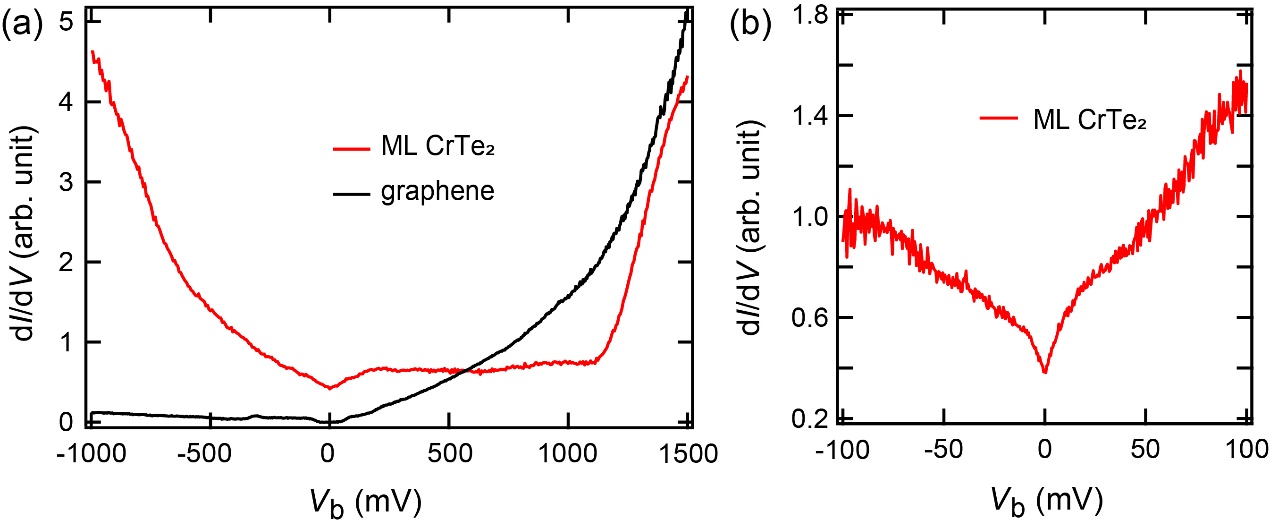


**Figure S2┃Spin-averaged tunneling spectra of ML CrTe_2_ and graphene.** (a) Large energy scale spectra (*V*_b_ = 1.5 V, *I*_t_ = 100 pA, *V*_mod_ = 14.14 mVrms) of ML CrTe2 and graphene measured with a W tip. (b) Small energy scale spectrum (*V*_b_ = 0.1 V, *I*_t_ = 100 pA, *V*_mod_ = 3.536 mV_rms_) of ML CrTe_2_ around the Fermi level.

**
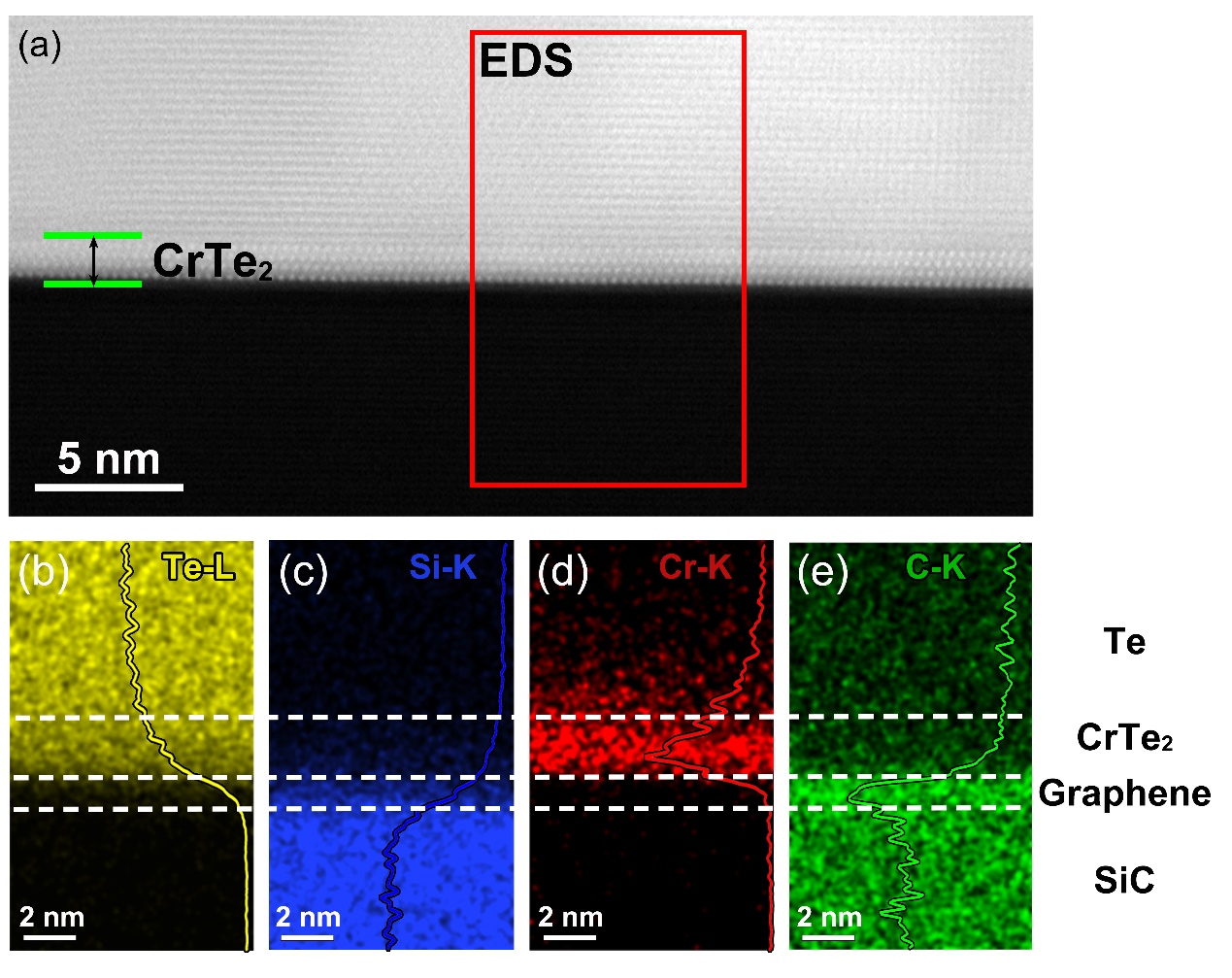
**

**Figure S3┃Elemental analysis of 2 ML 1T-CrTe_2_ films on the graphene/SiC surface.** (a) HAADF-STEM image of the side view of 2 ML 1T-CrTe_2_ film grown on the graphene/SiC surface. (b–e) EDS mapping and corresponding line profile of the red rectangle in (a). The Cr:Te ratio is calculated as 1:2.28 in the 2 ML 1T-CrTe_2_. The small deviation in the elemental composition from CrTe_2_ may originate from the upper Te layer and the weak elemental diffusion in the interface.


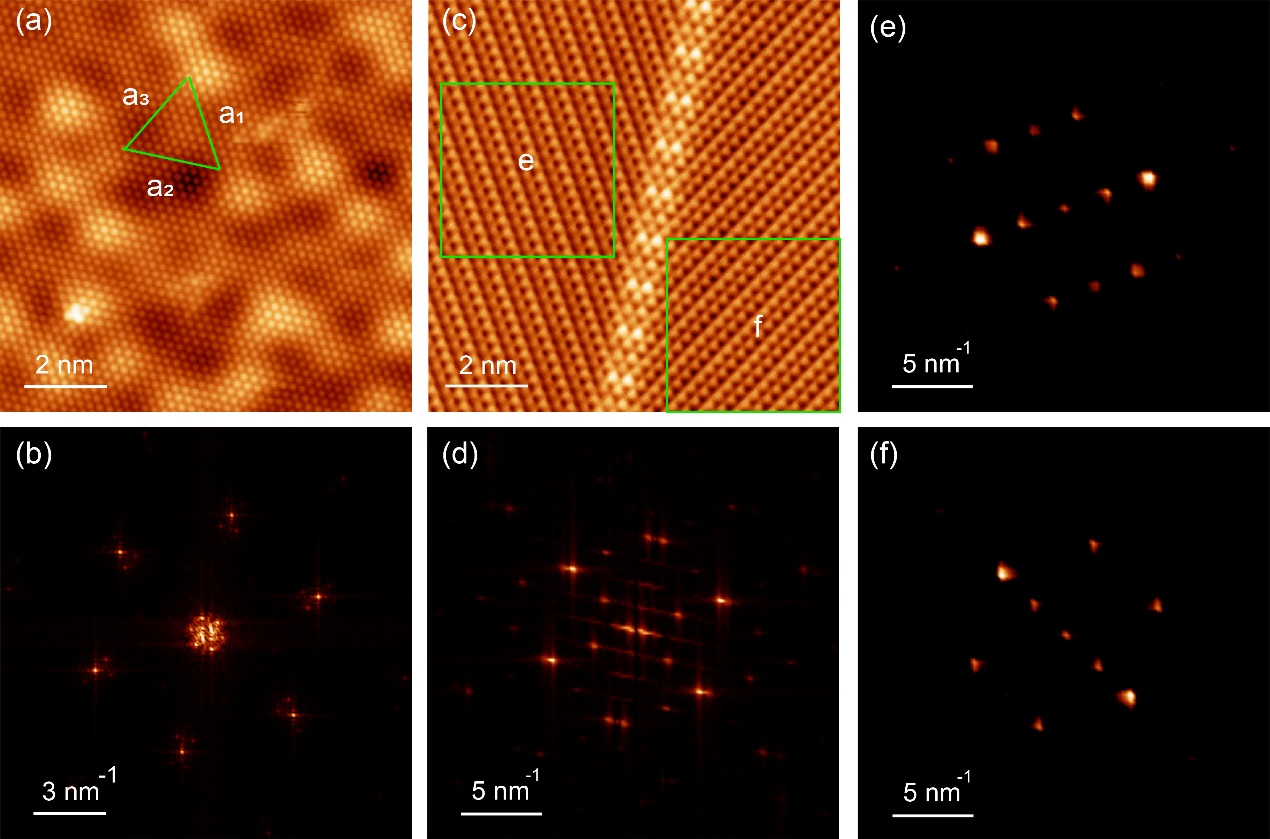


**Figure S4┃Atomic resolution of graphene and monolayer CrTe_2_.** (a,b) STM image (*V*_b_ = -100 mV, *I*_t_ = 500 pA) showing the atomic resolution of graphene (a) and its FFT image (b). The lattice units of graphene are indicated with a green triangle in (a). The imaged graphene lattices are measured as a_1_= 2.46 Å, a_2_= 2.45 Å, a_3_=2.46 Å, in good agreement with the theoretical value of 2.458 Å. (c,d) STM image (*V*_b_ = -500 mV, *I*_t_ = 10 pA) showing the atomic resolution of monolayer CrTe_2_ with a domain boundary (c) and its FFT image (d). The FFT pattern of (d) evidently contains two sets. (e,f) FFT images of the rectangle areas in (c), which changes to one set on each domain. The lattice constants of ML CrTe_2_ determined on domain of (e) are a_1_= 3.41 Å, a_2_= 3.72 Å, a_3_ = 3.73 Å. They are similar to those of (f), namely, a_1_= 3.45 Å, a_2_= 3.71 Å, a_3_ = 3.68 Å. Since the two domains are imaged with the exactly same condition, the lattice distortions of CrTe_2_ are unambiguously confirmed. The images in (a) and (c) are the raw data without filtering.


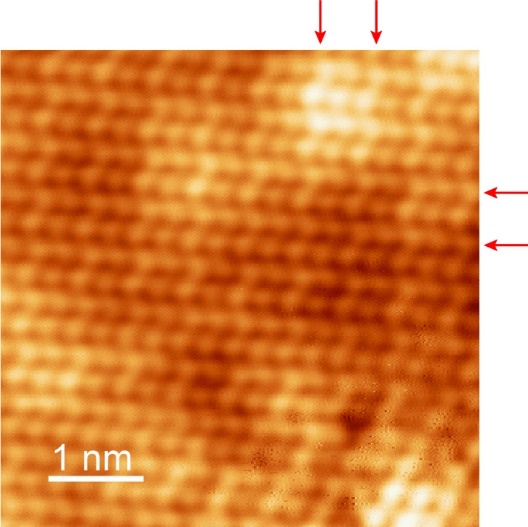


**Figure S5 ┃STM image of the atomic resolution of the ML CrTe_2_ taken with Cr-tip.** It confirms that the stripe direction of the 2×1 reconstruction (marked with horizontal arrows) and the zigzag structure (marked with vertical arrows) are orthogonal to each other. Imaging condition: *V*_b_ = −60 mV, *I*_t_ = 100 pA.


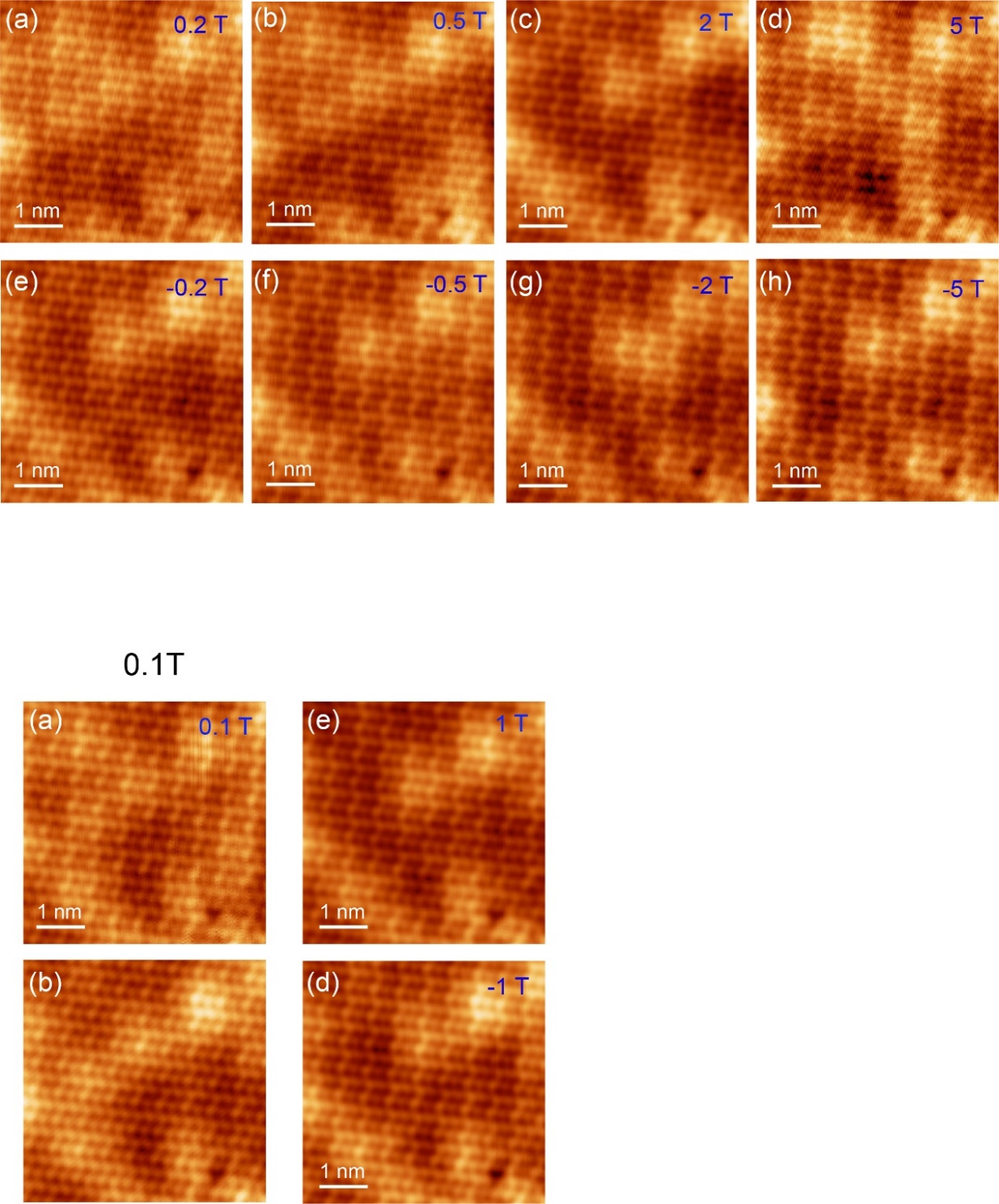


**Figure S6 ┃ SPSTM images of the same area as Fig. 2(a) under different magnetic fields.** Measurement conditions of (a–h): *V*_b_ = −0.1 V, *I*_t_ = 100 pA. The tip magnetization was kept unchanged during the measurements. For well-prepared Cr tips, the tip magnetization can sustain to high magnetic fields, e.g. 8 T in Ref. S4.


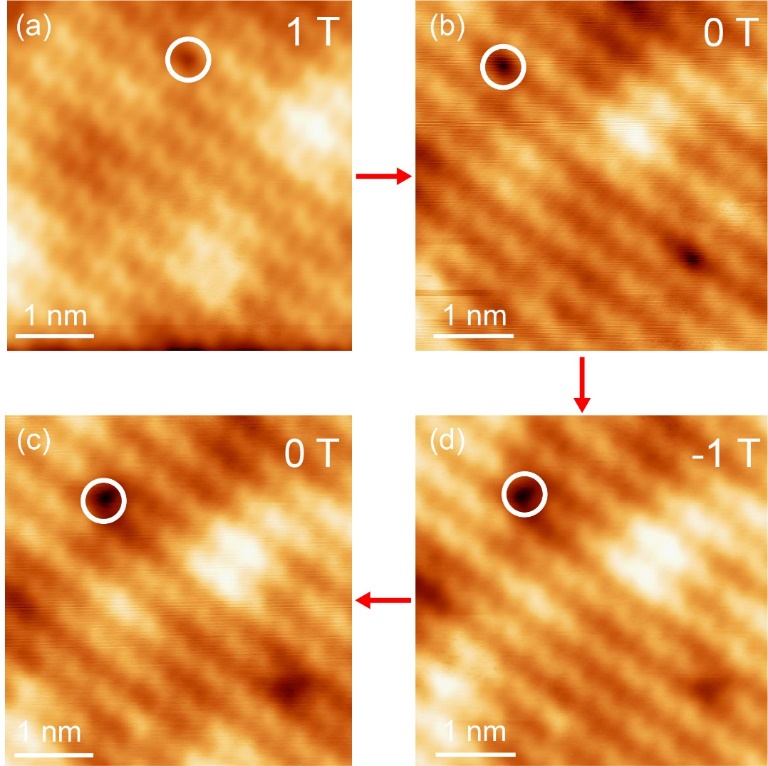


**Figure S7 ┃SP-STM images of 1 ML 1T-CrTe_2_.** The SPSTM images of the same region at the sequentially applied magnetic field. The white circles indicate a defect marker. For the spin contrast imaged at 0 T of (b) and (c), their spin contrast changes its phase, as is seen from the defect marker in white circle. This reflects the spin contrast is related to the history of the applied magnetic field. Measurement conditions of (a–d): *V*_b_ = 0.5 V, *I*_t_ = 100 pA.


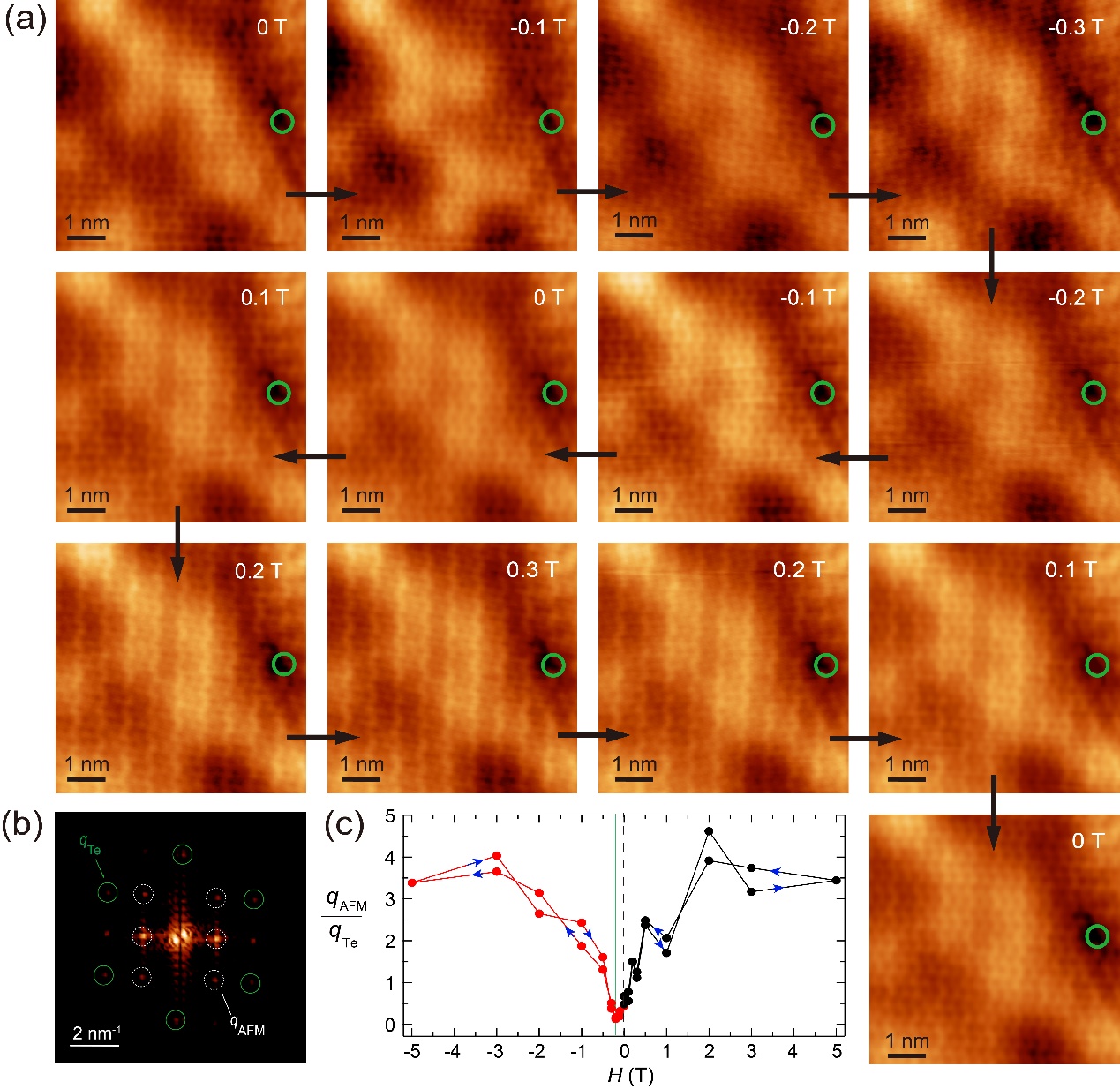


**Figure S8. Spin contrast variation of monolayer CrTe_2_ under magnetic fields.** (a) SPSTM images (*V*_b_ = 30 mV, *I*_t_ = 200 pA) of the same location of monolayer CrTe_2_ at different small magnetic fields, showing a spin contrast reversal field of -0.2 T. A defect marker is marked with a green circle in each image. (b) FFT image of SPSTM on monolayer CrTe_2_ at 1 T. The diffraction spots from the Te lattice (*q*_Te_) and the AFM spin contrast (*q*_AFM_) are shown. (c) FFT intensity of *q*_AFM_ relative to *q*_Te_ under different magnetic fields. The FFT intensities are extracted from the SPSTM images, where part of the images is shown in (a). The spin contrast intensity can be evaluated by extract from the ratio of *q*_AFM_ / *q*_Te_, namely, a more enhanced spin contrast produces a larger ratio in its corresponding FFT image. The data for positive (negative) fields is presented in black (red). The zero field and the spin-contrast reversal field of -0.2 T are marked with a black dashed line and a green line, respectively. The history of magnetic field application is depicted with black (blue) arrows in (a) [(b)].


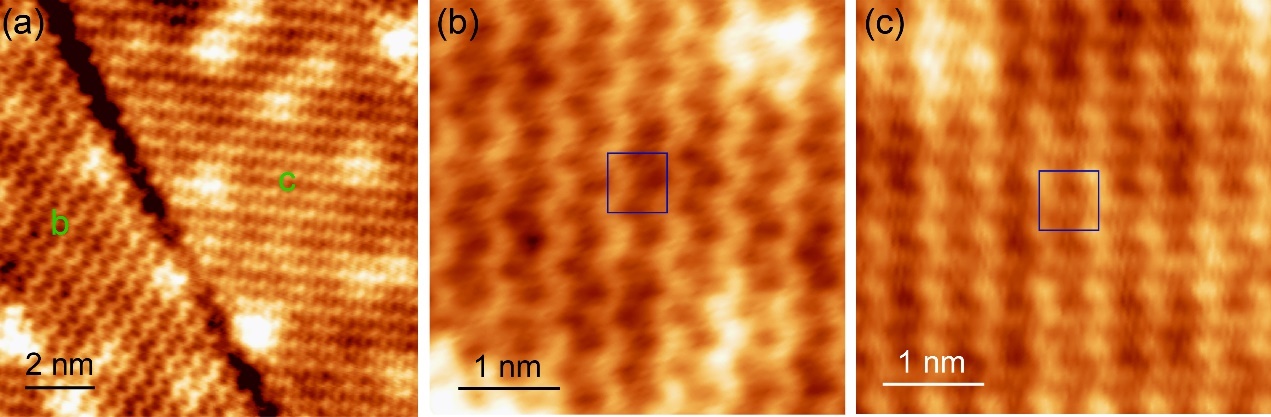


**Figure S9 ┃SPSTM image of different domains on the 1 ML 1T-CrTe_2_ surface with a Cr tip.** (a) Large-scale SPSTM image (*V*_b_ = +0.2 V, *I*_t_ = 100 pA) of ML CrTe_2_ showing a domain boundary. The structural domain coincides with the magnetic domain. (b,c) Zoom-in images of (a), which are extracted from the left (b) and right (c) domain. Note that the images of (b) and (c) have been rotated for comparison. The blue rectangles mark the AFM unit cell of the ML CrTe_2_. The spin-contrast of (b) is zigzag-like, while that of (c) is more rectangular-like. The spin contrast of the different domains is not identical, demonstrating a finite in-plane component of the canted tip magnetization, as is schematically shown in Fig. 3(h–j).


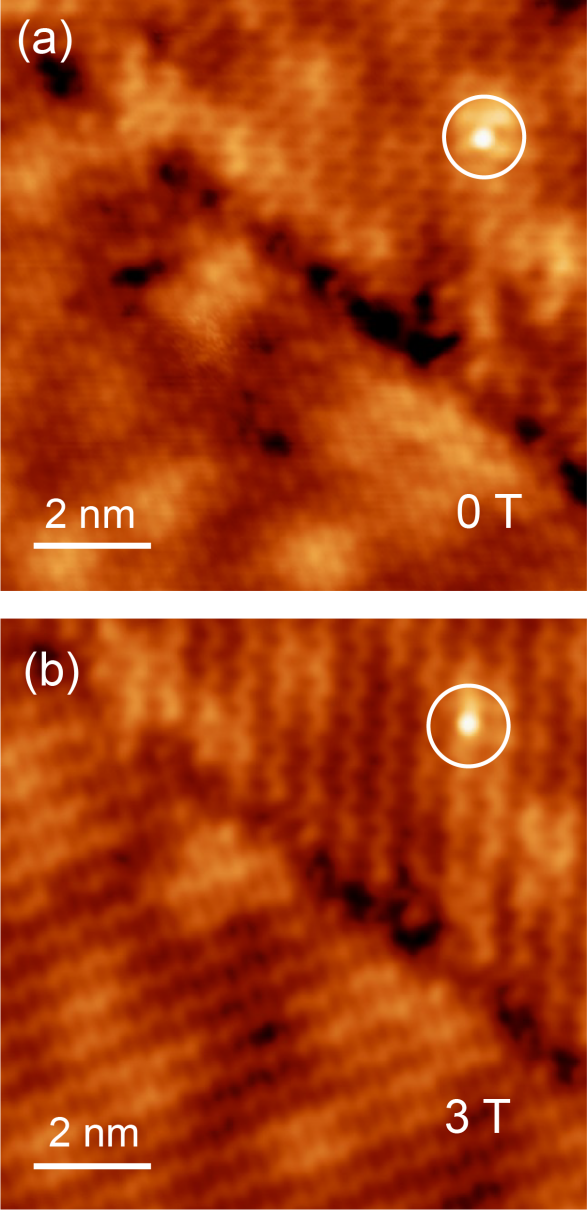


**Figure S10┃Spin-resolved images of ML CrTe_2_ taken with the Fe-coated tip.** The Fe-coated tips always provide in-plane spin sensitivity. The lower-left and upper-right domains show different spin contrasts in (a), demonstrating the in-plane spin component of the ML CrTe_2_. Under an out-of-plane field of 3 T (b), the spin contrast of both domains show a response to the field, and the upper-right domain switches its phase to a defect marker (white circle). This effect conforms to the conclusion that the Cr-coated tip in Fig. S9 has an in-plane component of spin sensitivity. Imaging conditions of (a and b): *V*_b_ = −1 V, *I*_t_ = 100 pA.


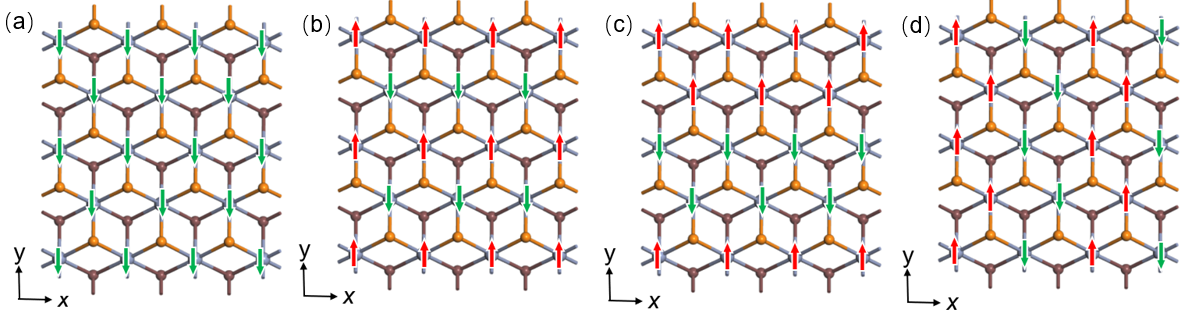


**Figure S11 ┃ Top views of schematics showing intralayer magnetic orders, including (a) FM, (b) sAFM-ABAB, (c) sAFM-AABB, and (d) zigzag in the ML CrTe_2_.** Light-slate-grey, orange, and brown balls represent the Cr atoms, top Te atoms, and bottom Te atoms, respectively. Green and red arrows denote the magnetization directions of Cr atoms.


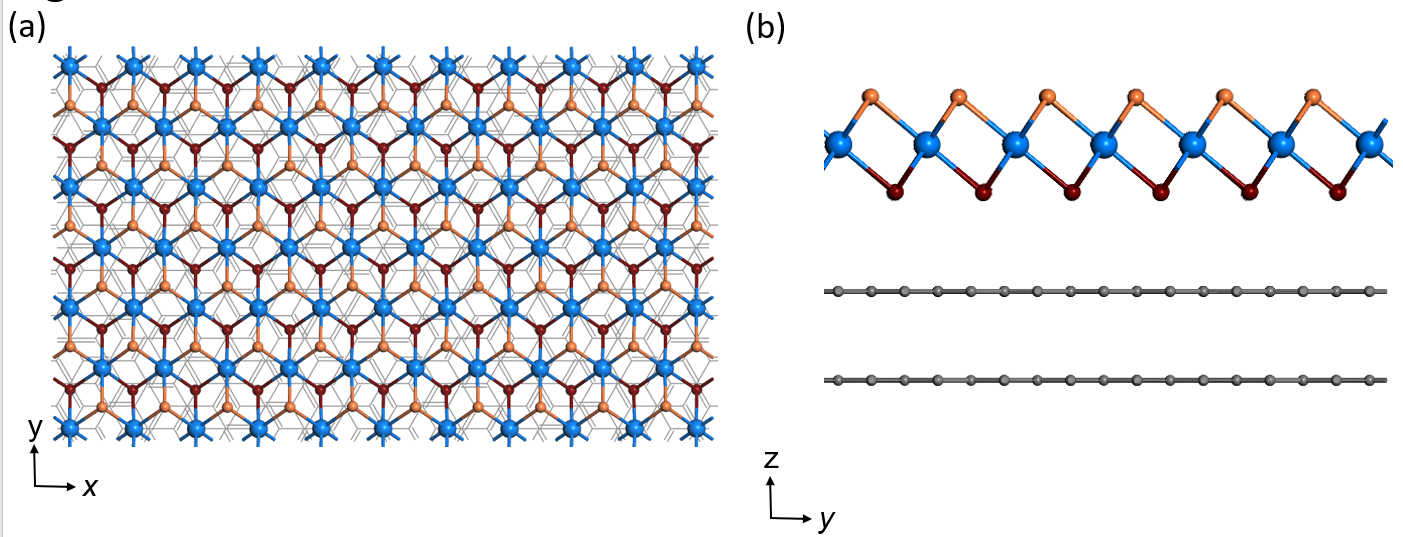


**Figure S12 ┃ Top and side views of the 10×3ML CrTe_2_ on the 16×4bilayer graphene heterostructure model.** The grey lines correspond to the graphene substrate in the top view for clarity. The experimental lattice constants are a_1_= 3.4 Å, a_2_= 3.7 Å, a_3_ = 3.7 Å. The fully relaxed lattice a_1_= 3.6 Å, a_2_= 3.6 Å, a_3_ = 3.7 Å in our calculations of free standing CrTe_2_ monolayer suggests a substrate induced in-plane compressive strain of 5% along a_1_ and tensile strain of 3% along a_2_, which could be modeled in a heterostructure of 10×3CrTe_2_ stacking on 16×4bilayer graphene. We thus double checked our conclusions with the revised lattice and hetero-model (Table S1 and S2) and found our previously believed magnetic ground state of the zigzag (ZZ) order is still valid with even more pronounced stability.


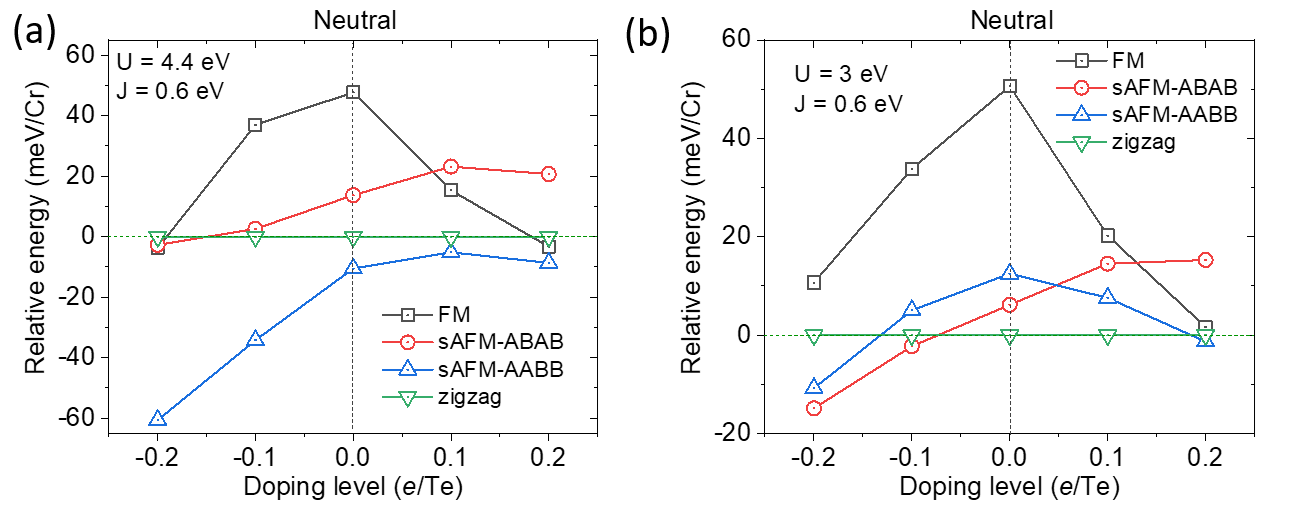


**Figure S13 ┃** Relative total energies of the zigzag (green reverse triangles), sAFM-AABB (blue triangles) and sAFM-ABAB (red circles) orders as a function of the electron/hole doping level using U = 4.4 eV (a) and 3.0 eV (b) calculated with optB86b-vdW+UJ. The zigzag order was chosen as the reference zero. By using a more reasonable U value of 3 eV, our calculations indicate the zigzag order has the lowest total energy in a wide range of doping levels and should be robust in our experiments.


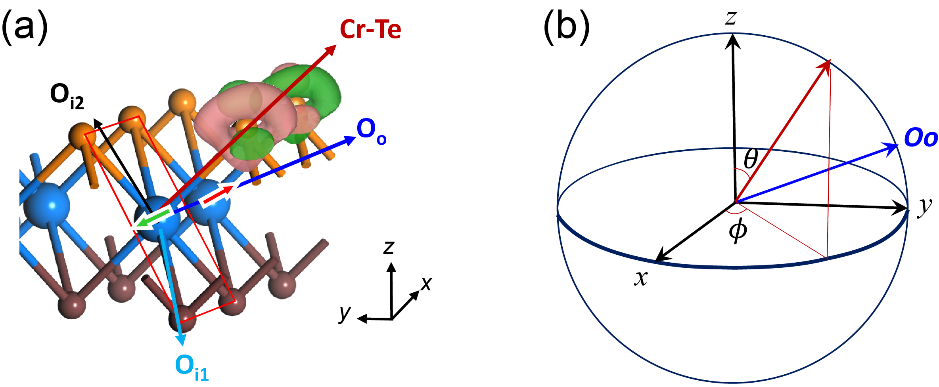


**Figure S14 ┃ Magnetization axes considered in the calculation of the magnetic anisotropy energy.** Here, angles 𝜃 and *ϕ* correspond to the angles between the magnetization direction and the *z* and *x* axes, respectively.


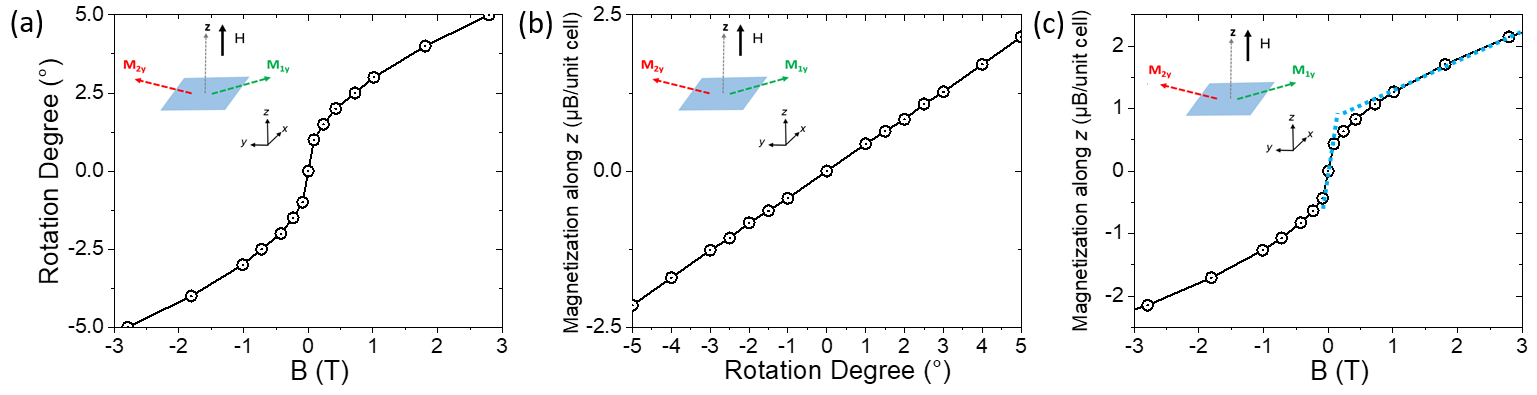


**Figure S15 ┃ The rotation of magnetic moments under out-of-plane field.** (a) Theoretical estimated rotation degree of magnetizations moments direction under magnetic field from -3 T to 3 T. (b) The net magnetization along *z* as a function of the rotation degree of magnetic moments. (c) Evolution of the net magnetization along *z* under external magnetic field. Blue dashed lines represent the slope of the net magnetic moments. We carried out calculations to examine the angular dependent relative energies of the non-collinear orders, which gives the relationships of rotation angle under magnetic field (a) and of magnetic moment with respect to rotation angle (b). Both plots derive a more detailed evolution relation between magnetic moment and magnetic field (c).


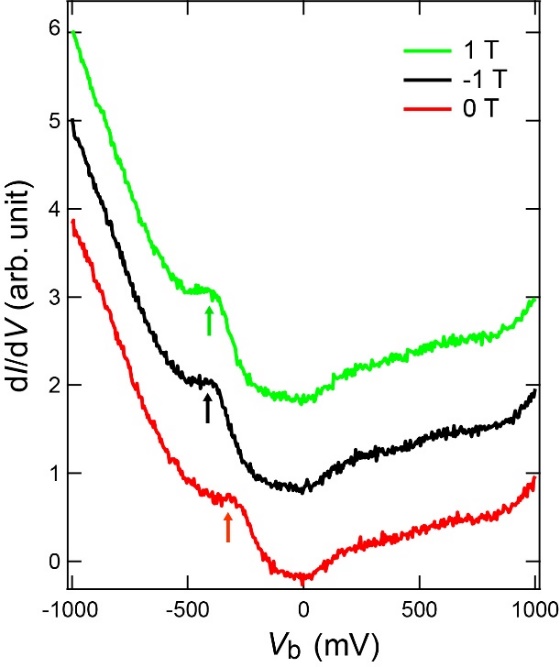


**Figure S16 ┃ Spin-resolved spectra of ML CrTe_2_ at various magnetic fields taken with a different micro Cr tip to that of Fig. 4(e).** The arrows mark the energy evolution of the peak with the magnetic field. The peak energies fitted with Lorentz shapes are *E*(1T) = -388.56 meV, *E*(-1T) = -384.29 meV, *E*(0T) = -290.94 meV. Those values are *E*(1T) = -564.22 meV, *E*(-1T) = -535.93 meV, *E*(0T) = -479.22 meV in Fig. 4(e). While the specific peak energies at corresponding magnetic fields are not identical with the different micro Cr tips, their evolution trend is the same. Spectroscopic conditions: *V*_b_ = −1 V, *I*_t_ = 100 pA, *V*_mod_ = 14.14 mV_rms_.


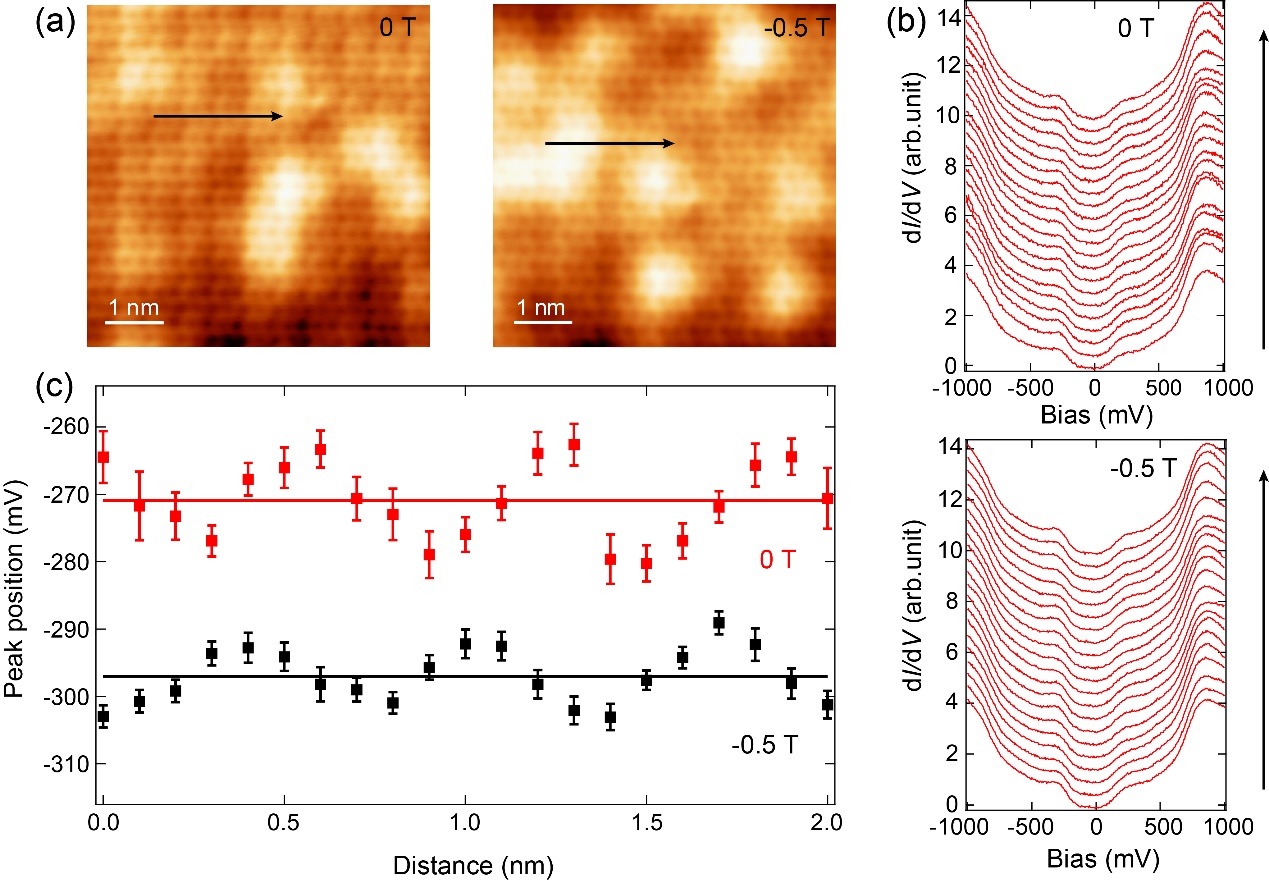


**Figure S17┃ Spatial variation of the peak energy.** (a) SPSTM images (*V*_b_ = −0.2 V, *I*_t_ = 100 pA) of the 1 ML 1T-CrTe_2_ film on the same area at the magnetic field. (b) Series of tunnelling spectra (*V*_b_ = 1 V, *I*_t_ = 100 pA, *V*_mod_ = 7.07 mV_rms_) taken along the lines of (a). (c) Energy of peak in (b) obtained by fitting with a Lorentz shape and a polynomial background. There is a spatial variation of the peak energy, which correlates with the zigzag magnetic pattern. Note that the micro Cr tip measured in this set of data is different to that in Figs. 2 and 4. There are some bright spots in (a), which are from the 6×6 reconstruction between the graphene and SiC interface. They do not affect the peak around -0.5 V, as is seen in (c).


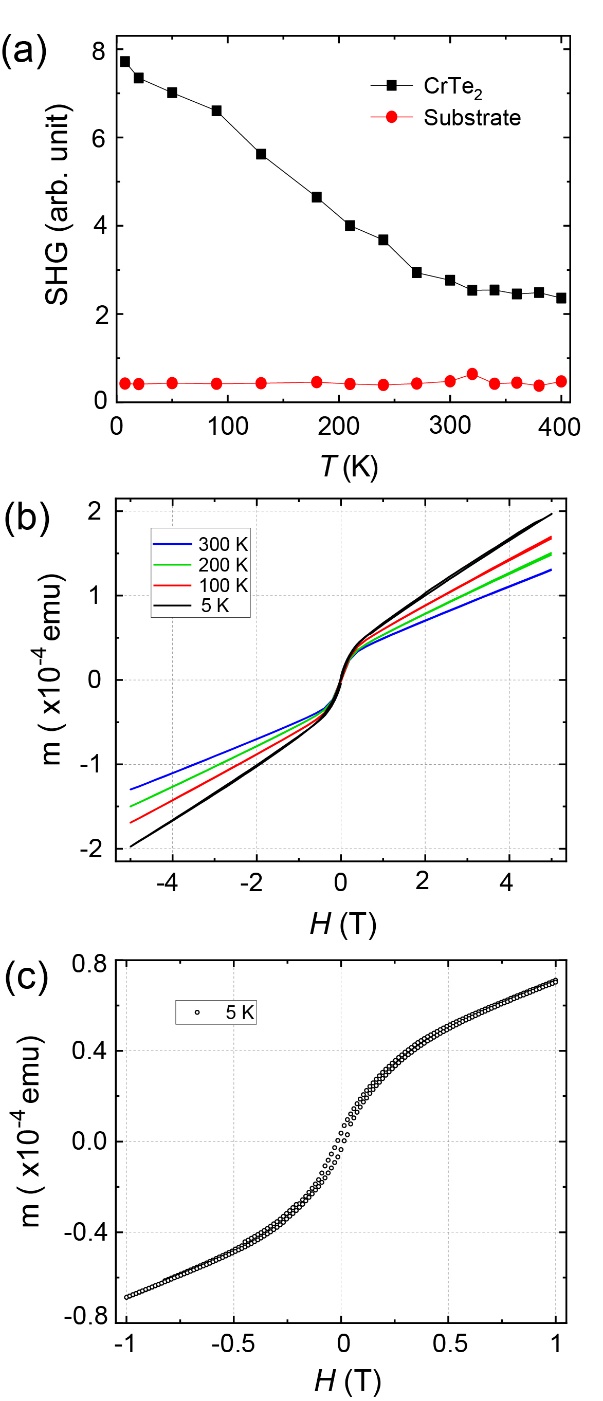


**Figure S18┃ SHG and SQUID measurements on ML CrTe_2_.** (a) Temperature dependent SHG measurements of ML CrTe_2_ and the substrate. (b) Out-of-plane *m*-*H* curves of ML CrTe_2_ taken with SQUID at different temperatures, showing AFM order. (c) Out-of-plane *m*-*H* curves of ML CrTe_2_ taken with SQUID at 5 K, showing spin-flop transition.

**Table S1 ┃**Geometric and magnetic details of the free-standing and substrate-supported ML CrTe_2_.

| CrTe2-1L | | Mag. Config. | △E  (meV/Cr) | a(Å) | Mag. Mom. (μB) | |
| --- | --- | --- | --- | --- | --- | --- |
|  |  |  |  |  | Cr | Te |
| Free standing | Fully relaxed | FM | 25.5 | 3.7/3.7/3.7 | 3.11 | -0.18 |
|  |  | sAFM-ABAB | 6.0 | 3.5/3.7/3.7 | 3.03 | -0.04 |
|  |  | sAFM-AABB | 4.4 | 3.5/3.6/3.7 | 3.16 | -0.06 |
|  |  | Zigzag | 0.0 | 3.6/3.6/3.7 | 3.08 | -0.04 |
|  | Hetero. Lattice | FM | 76.8 | 3.4/3.7/3.7 | 3.13 | -0.15 |
|  |  | sAFM-AABB | 59.9 |  | 3.18 | -0.14 |
|  |  | Zigzag | 0.0 |  | 3.08 | -0.04 |
| On bilayer graphene substrate | Hetero. Lattice | FM | 71.0 | 3.4/3.7/3.7 | 3.15 | -0.18 |
|  |  | Zigzag | 0.0 |  | 3.07 | -0.06 |

**Table S2 ┃**Magnetic anisotropy energies (MAE) of the free-standing and substrate-supported ML CrTe_2_ in the zigzag order. The magnetization axes listed here are marked in Fig. S14a. The substrate effect would not change the easy axis direction.

| CrTe2-1L-Zigzag | Mag. Axis | MAE  (meV/Cr) |
| --- | --- | --- |
| Free standing | *x* | 0.83 |
|  | *y* | 0.16 |
|  | *z* | 1.96 |
|  | *O*_o_ | 0.00 |
|  | *O*_i1_ | 0.80 |
|  | *O*_i2_ | 1.53 |
| On  Bilayer  Graphene | *x* | 0.74 |
|  | *y* | 0.31 |
|  | *z* | 2.16 |
|  | *O*_o_ | 0.00 |
|  | *O*_i1_ | 1.14 |

Table S3. K-mesh convergence of MAE of free-standing ML CrTe_2_ based on constrained lattice constants

| CrTe_2_-1L-Zigzag | Mag. Axis | MAE  (meV/Cr) | | | |
| --- | --- | --- | --- | --- | --- |
|  |  | 10×6×1 | 14×8×1 | 18×10×1 | 20×12×1 |
| Free standing | *x* | 0.83 | 0.92 | 0.87 | 0.90 |
|  | *y* | 0.16 | 0.13 | 0.12 | 0.12 |
|  | *z* | 1.96 | 1.91 |  |  |
|  | *O*_o_ | 0.00 | 0.00 | 0.00 | 0.00 |
|  | *O*_i1_ | 0.80 | 0.82 |  |  |
|  | *O*_i2_ | 1.53 | 1.45 |  |  |

**SUPPLEMENTARY REFERENCES**

S1. Wiesendanger, R. Spin mapping at the nanoscale and atomic scale. *Rev. Mod. Phys.* **81**, 1495 (2009).

S2. Fu, Y.S., Xue Q.K., Wiesendanger, R. Spin-Resolved Splitting of Kondo Resonances in the Presence of RKKY-Type Coupling. *Phys. Rev. Lett.* **108**, 087203 (2012).

S3. Schwöbel, J. et al. Real-space observation of spin-split molecular orbitals of adsorbed single-molecule magnets. *Nat. Commun*. **3**, 953 (2012).

S4. Khajetoorians, A.A. *et al*. Detecting excitation and magnetization of individual dopants in a semiconductor. *Nature* **467**, 1084–1087 (2010)
